# Perspectives from the Third International Summit on Medical Nutrition Education and Research

**DOI:** 10.3389/fpubh.2018.00093

**Published:** 2018-03-23

**Authors:** Jennifer Jean Crowley, Celia Laur, Harrison David Edward Carter, Glenys Jones, Sumantra Ray

**Affiliations:** ^1^Discipline of Nutrition, Faculty of Medical Health Sciences, University of Auckland, Auckland, New Zealand; ^2^NNEdPro Global Centre for Nutrition and Health, St John’s Innovation Centre, Cambridge, United Kingdom; ^3^Faculty of Applied Health Sciences, School of Public Health and Health Systems, University of Waterloo, Waterloo, ON, Canada; ^4^Department of Public Health and Primary Care, School of Clinical Medicine, Institute of Public Health, University of Cambridge, Cambridge, United Kingdom

**Keywords:** nutrition, public health, health care, policy, global food systems, implementation

## Abstract

Nutrition is an important component of public health and health care, including in education and research, and in the areas of policy and practice. This statement was the overarching message during the third annual International Summit on Medical Nutrition Education and Research, held at Wolfson College, University of Cambridge, United Kingdom, in August 2017. This summit encouraged attendees to think more broadly about the impact of nutrition policy on health and communities, including the need to visualize the complete food system from “pre-farm to post-fork.” Evidence of health issues related to food and nutrition were presented, including the need for translation of knowledge into policy and practice. Methods for this translation included the use of implementation and behavior change techniques, recognizing the needs of health-care professionals, policy makers, and the public. In all areas of nutrition and health, clear and effective messages, supported by open data, information, and actionable knowledge, are also needed along with strong measures of impact centered on an ultimate goal: to improve nutritional health and wellbeing for patients and the public.

## Introduction

**Figure 1:**
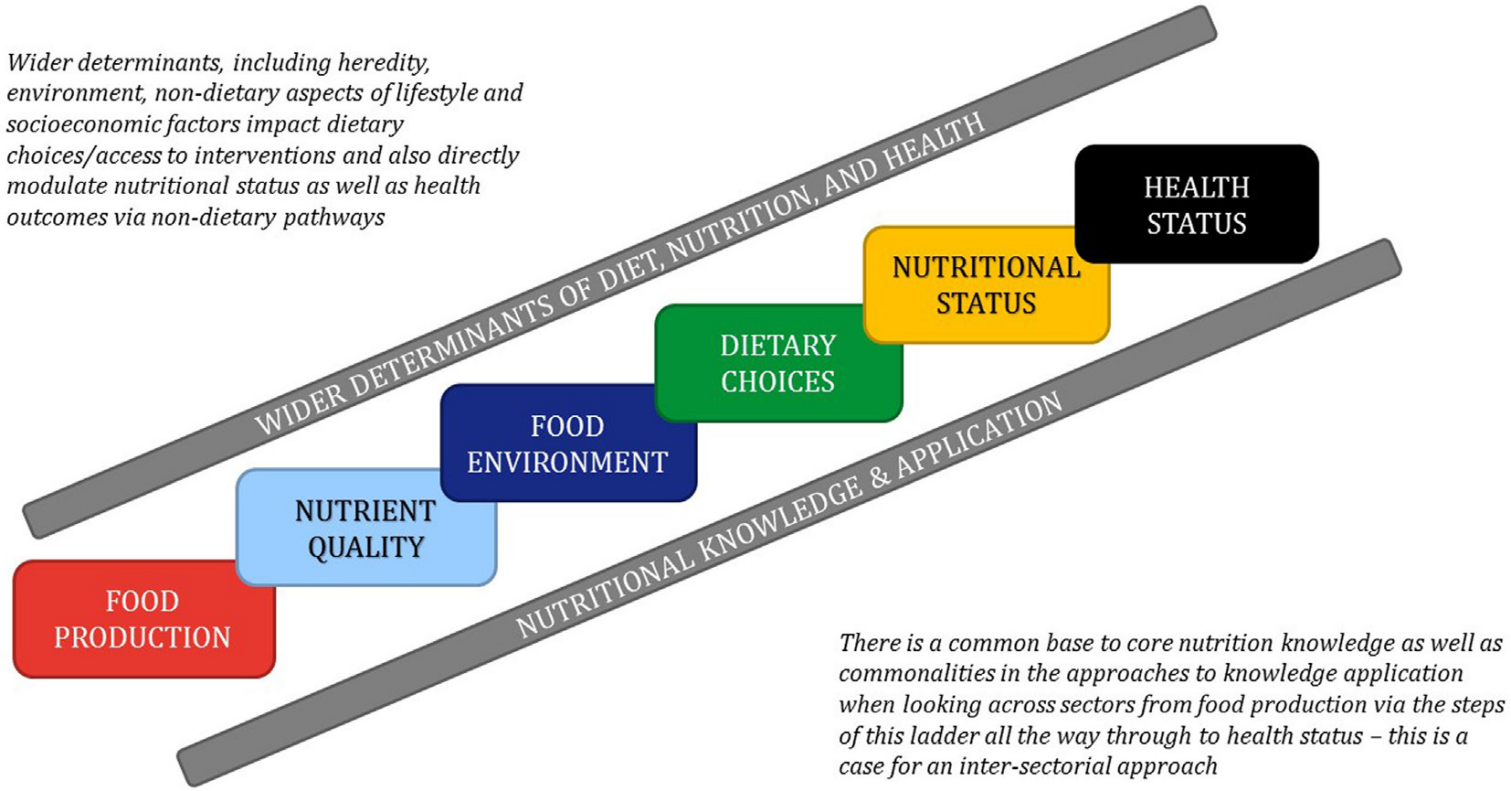
A schematic representation of the conference themes.

Nutrition has a vital role in maintaining health and preventing disease. As such, a chain of events from food production, through the food environment to dietary choices, advice and interventions, summatively impact nutritional status, thus modulating health or disease. With an increasing recognition of the preventative role of nutrition in health-related policy and practice, effective strategies are needed to bridge current divides between nutrition research and professional education, as well as between the agricultural and human health-related knowledge bases in nutrition. This was the main message of the third annual International Summit on Medical Nutrition Education and Research event hosted by the Need for Nutrition Education/Innovation Programme (NNEdPro) Global Centre for Nutrition and Health, and the Global Open Data for Agriculture and Nutrition (GODAN) at Wolfson College, University of Cambridge, United Kingdom (UK) on August 1–2, 2017. The Summit brought together international organizations and individuals involved in nutrition education and research. This year, the focus was on how to work together to build a strong evidence base and translate that evidence into policy and practice, looking at the whole system of food, nutrition, and health. A schematic representation of the conference theme, as outlined by Sumantra Ray (SR) and André Laperrière (AL) is provided in Figure 1, and a list of speakers and key messages is in Table 1. The aim of this perspective is to outline key messages from the Summit and present ideas for future consideration.

**Table 1:** Speakers and key messages from the Third International Summit on Medical Nutrition Education and Research.

Speaker name	Organization	Country	Key message
**Day 1 (AM): engagement with policy makers and commissioners**			

**Moderators: Dr. Jennifer Crowley^a^ and André Laperrière^a^**			
Dr. Francesco Branca	World Health Organization (WHO)	Switzerland	Policy coherence across food systems and agriculture is required to address the double burden of disease that exists throughout the world to enable the United Nations to achieve the Sustainable Development Goals-agenda-2030

Boshko Stankovski^a^	Trinity College, University of Cambridge	Republic of Macedonia	The evolving nature of the international right to food has relevance to nutrition research and education

Professor Chris Baker	IPSNP Computing Inc. (Canada, UK), University of New Brunswick, Canada	Canada	Real world decision-support (as outlined by the Food and Agriculture Organization of the United Nations “Use Cases”) requires rapid access to numerous distributed sources and analysis of data. Queries to these diverse and heterogeneous data sources are typically *ad hoc* in nature. A unique advanced search engine, HYDRA, is able to discover and retrieve relevant sources of data online and integrate it such that the data can provide comprehensive answers. Examples of queries requiring the federation and integration of data were in support of decisions on: which varieties of eggplant to grow, the corresponding profit margins, and consequences of crop husbandry (pesticides) on non-target organisms (bees)

Dr. Guiseppe Grosso^a^	Integrated Cancer Registry, Azienda Ospedaliera Universitaria Policlinico Vittorio Emanuele	Italy	Strong evidence is required to inform public health messages when researching the impact of food and nutrition in humans

Dr. John Ingram	University of Oxford	UK	Food systems can provide the link between food production and good nutrition, including food security and nutrition security, when there is good communication between plant breeders, agronomists, farmer education, and public health messaging

Kiringai Kamau	University of Nairobi	Kenya	Data driven, student led, agricultural extension models can align infrastructure to support the governments’ efforts to address producer community knowledge delivery gaps

Professor Louis Levy	Public Health England (PHE)	UK	Nutrition research fits into the policy cycle of identifying needs, options, implementation, monitoring, and evaluation review and requires evidence at each stage of the cycle

**Day 1 (PM): role of health-care providers in delivering nutritional care and nutrition skills in practice**			

**Moderator: Nida Ziauddeen^a^**			
Melissa Adamski	Monash University	Australia	Monash University has a suite of online courses, Food as Medicine. The Food as Medicine MOOC is an educational tool aimed at the general public, which provides free, flexible online learning in areas of food, nutrition, and health. The suite of for-fee flexible online courses are for HCP to provide relevant and useful nutrition information that can be used in health-care practice

Celia Laur^a^	University of Waterloo	Canada	NNEdPro’s Global Innovation Panel supports individuals and groups to enhance the skills of HCP and translate evidence into practice

Dr. Jennifer Crowley^a^	University of Auckland	NZ	The Australia New Zealand Network supports medical nutrition educators progress medical/health-care nutrition research
Professor Eleanor Beck^a^	University of Woollongong	Australia	

James Bradfield^a^	University College Cork	UK	NNEdPro has developed a multipronged educational strategy ranging from e-learning for health care and related professionals to educational interventions in poorly resourced settings. I-KANN-25 will serve as an international knowledge exchange platform to bring together existing knowledge and also provide a platform to share best practice within and across regions
Shivani Bhat^a^	NNEdPro	Canada	
Professor Sumantra Ray^a^	NNEdPro	UK	

Eritia Abulu	University of Hereford	UK	Student and health-care professionals illustrated different approaches to the key role pharmacists could provide at the interface between the public and HCP to deliver nutrition advice to patients
Miranda van Emmenis	University of Cambridge	UK	
Pauline Douglas^a^	University of Ulster	UK	
Candice Ward	Cambridge Diabetes Education Programme (CDEM)	UK	

Dr. Robert Winwood	Royal DSM	UK	Some drug nutrient interactions adversely affect micronutrient status in human tissue, while others can benefit micronutrient interactions

Selvarani Elahi^a^	Laboratory of the Government Chemist (LGC)	UK	The Food Authenticity network is a virtual initiative to bring together those involved in food authenticity testing across the UK, and in other countries access to a network of food authenticity testing laboratories

**Day 1 (mini symposium): translating novel data on polyphenols onto practice**			

**Moderator: Professor Daniele Del Rio^a^**			
Dr. Christina Khoo	Ocean Spray	USA	Ocean Spray is associated with universities and government agencies to understand the nutritional and health benefits of the cranberry

Dr. Daniela Martini	University of Parma	Italy	The three main pillars used to judge health food claims provide guidance for the research required to have claims substantiated

Dr. Donato Angelino	University of Parma	Italy	Emerging evidence suggests that polyphenols, particularly procyanidins, found in tea, cocoa, grapes, nuts, and berries are beneficial for cognitive function

Professor Nathalie Tufenkji	McGill University	Canada	Emerging evidence suggests that cranberry may be an alternative to use of antibiotics for the prevention and treatment of infections

Geoffrey Istas	Kings College London	UK	Emerging evidence suggests that cranberry polyphenols provide beneficial effects to the vascular health of healthy men

Professor Kalpana Gupta	Boston University School of Medicine	USA	Recent research demonstrates that cranberry lowers urinary tract infections in women

**Day 2: Implementation science-moving research into practice**			

**Moderators: Dr. Margaret Ashwell (AfN), Dr. Glenys Jones^a^, Professor Martin Kohlmeier^a^**			
Celia Laur^a^	University of Waterloo	Canada	Finding effective ways of getting evidence into routine practice using implementation science and practice. Examples from the More-2-Eat implementation project

Harrison Carter^a^	University of Cambridge	UK	Practicable solutions are being sought to address barriers that challenge implementation policy to address hospital malnutrition

Pauline Douglas^a^	NNEdPro	UK	The cow’s milk allergy project aims to bring best practice from hospital care into primary care and community care practice
Dr. Glenys Jones^a^	NNEdPro	UK	
Dr. Minha Rajput Ray^a^	NNEdPro	UK	

Associate Professor Suzanne Piscopo^a^	Society for Nutrition Education and Behavior (SNEB)	Malta	To promote healthy eating behaviors that are effective, interventions need to be based on behavior change theory, target specific audiences, and go beyond disseminating information to nurturing a willingness to improve food intake and creating an enabling environment
	University of Malta		

Dr. Laura Thomas	Laura Thomas Ph.D.	UK	Intuitive eating principles can create behavior change in practice
	“Don’t Salt My Game”		

Victor Mogre	University for Development Studies, Tamale	Ghana	Educational interventions should emphasis building skills, self-efficacy, and role modeling of nutrition care by leaders in the practice setting to improve nutrition practice behavior. Future studies should measure improved clinical outcomes that result from change in nutrition practice behavior

Victoria Avery	Yakult	UK	There is potential for probiotics, given in conjunction with an educational programme for care home staff, to counter the effects of age-related changes to gut microbiota, and enhance the health of care home residents

Anthony Warner	The Angry Chef	UK	“Fake news” related to nutrition illustrates the importance of evidence-based and easily understood nutrition communication and public health messaging

Dr. Giles Yeo	University of Cambridge	UK	Academics have a duty to stand up for and be passionate about the truth, but how it is told also matters

**Day 2: panel discussion: the creation of the nutrition data strategy**			

**Moderator: André Laperrière^a^**			

Ruthie Musker^a^	Global Open Data for Agriculture and Nutrition (GODAN)	UK	The need for and benefits of open access data were supported by most summit attendees who provided suggestions how NNEdPro and GODAN can continue to advocate for it/open access data
Nida Ziauddeen^a^	University of Southampton	UK	
Dr. Glenys Jones^a^	NNEdPro	UK	

*^a^NNEdPro Member*.

*GTA, Global Training Academy; HCP, health-care professionals; NELICO, Nutrition Education and Leadership for Improved Clinical Outcomes; NZ, New Zealand; MOOC, Massive Open Online Course; USA, United States of America*.

## Impacting Nutrition with a Food Systems Approach

While nutritional issues can be approached from different perspectives when considering individual United Nations (UN) Sustainable Development Goals (SDG), the UN Decade of Action on Nutrition aims to bring together a matrix of SDG-relevant actions with Nutrition as a common denominator (1, 2). Considering a food systems approach, it is recognized that well-designed policy can impact “pre-farm” by influencing food production from the very beginning of the food-cycle. Determining what crops farmers grow can impact on the farming methods needed to tend and nurture the crop, as well as influence resultant yields. Farming practices also impact food production, which impacts on food environment and nutrient quality, thus subsequently influencing dietary choices, leading to the “post-fork” impact on nutritional and health status. Examining this whole systems approach demonstrated the need for strong evidence and ways to translate that evidence into policy and practice at all levels within the system, including clear communication, and public health messaging.

## Engaging Policy Makers and Commissioners to Improve Health

Engaging with policy makers and commissioners is essential, particularly when looking at improving health outcomes of individuals by taking a systems approach of “pre-farm to post-fork.” In line with this approach, Franceso Branca from the World Health Organization (WHO) outlined priorities for global nutrition policy in the context of the SDG—agenda 2030, particularly Goal 2: *End hunger, achieve food security, and improved nutrition and promote sustainable agriculture* (1). Policy makers should be addressing the double burden of malnutrition, as undernutrition or overweight/obesity, combined with nutrition-related non-communicable diseases (NCD), can all play a role at individual, household, and population levels, and across the lifespan. Policy coherence is needed across food systems and agriculture, and the UN Decade of Action on Nutrition (2) demonstrates that nutrition is a key concern for the WHO commission.

Policy makers should also recognize that access to open data can support policy decisions in agriculture. For example, Chris Baker presented a search tool that provides a user-friendly graphical interface, for complex *ad hoc* query composition suitable for policy makers or programme managers. This tool will have a query engine that is able to discover online data resources from multiple organizations (e.g., the European Food Safety Authority), retrieve, and integrate the data on a per query basis. This tool could help determine the type of crops and quantities to be grown that would contribute to a healthier diet for a specific population demographic.

John Ingram discussed how policy makers should know the potential for food systems to bridge food production and nutrition. As part of this, they should recognize the differences between the Food and Agriculture Organization of the United Nations definition of “*food security,”* and the Committee on World Food Security (CFS) definition of “*nutrition security*.” Overall, “*nutrients are seen as a crucial component of security, and food security is seen as a crucial component of nutrition security*” (3, 4). A food system perspective is needed to structure the necessary dialog between researchers along the whole food chain (plant breeders, agronomists,^1^ extension services (farmer education),^2^ raw material processors, final product processors, through to nutritionists, anthropologists, and behavioral psychologists) to link agriculture to nutrition outcomes. Policy makers should also acknowledge the need to educate farmers about which crops to grow/produce, as discussed by farmer and agricultural economist, Kiringai Kamau.

From a cross-border legal perspective, the evolving nature of the international right to food, and its relevance within nutrition research and education was presented by Bosko Stankovski. Policy makers should know that the right to food is progressive and should be seen as dynamic, not static, as evidenced in three UN documents (5–7). Giuseppe Grosso discussed the need for strong evidence to inform policy and public health messages, recognizing the limitations of some studies, including the ethical challenges when researching the impact of food and nutrition in humans.

Louis Levy (LL) of Public Health England (PHE) presented a UK perspective on nutrition evidence, policy development, and implementation. LL discussed how nutrition research fits into the policy cycle of identifying needs and options, following through to implementation, monitoring, and evaluation/review. Evidence is required at each stage of the cycle and is accompanied by consultation when applicable.

## Health-Care Providers have a Key Role in Delivering Nutritional Care

To put nutrition policies into practice, one approach is to focus on educating health-care providers and developing their skills to deliver safe and effective nutrition care. For example, the Food as Medicine, Massive Open Online Course, presented by Melissa Adamski, provides free, flexible, online learning for the general public, with education on the relationship between food, nutrition, and health. Under this Food as Medicine brand, a suite of for-fee online courses have also been developed for health-care professionals (HCP) without a nutrition background, or as refresher courses for nutrition professionals (8), a number of which have been externally quality assured by the UK Association for Nutrition.

To further encourage international shared learning in nutrition education, the NNEdPro’s Global Innovation Panel (GIP) supports individuals and groups to work and learn together to enhance the skills of HCP and translate evidence into practice. For example, within GIP, the Australia and New Zealand Network (ANZ Network) aims to support medical nutrition educators progress medical/health-care nutrition research. NNEdPro is also developing e-learning materials for the University of Cambridge, School of Clinical Medicine, which is also included as pre-learning material for the NNEdPro Summer School Foundation Certificate Course in Applied Human Nutrition. The global application of such teaching methods was illustrated in NNEdPro’s Urban Slum Project that delivered a series of workshops to educate local HCP and lay volunteers on delivering nutrition advice to the urban slum population in India (9).

Another way that NNEdPro supports HCP education is through the International Knowledge Application Network in Nutrition 2025 initiative (I-KANN-25), which uses the global increase in NCDs as an example to illustrate its application. I-KANN-25 is part of NNEdPro’s education and training academy, which facilitates: nutrition education at the University of Cambridge; the Summer School in Applied Human Nutrition (Cambridge); the annual International Summit (Cambridge); and e-learning initiatives. I-KANN-25 seeks to connect materials from these initiatives and more, to be used internationally, such as through the development of an online portal, which will encourage regional adaptations and opportunities for international interaction to facilitate learning. The I-KANN-25 online network will be modeled on the Food Authenticity Network, developed by the Department for Environment, Food and Rural Affairs (Defra). The Defra initiative spans 21 countries to bring together those involved in food authenticity testing (10).

### The Role of Nutrition in Pharmacy

Pharmacists play a key role within primary care, often having more contact with members of the community than other HCP (11). For this reason, pharmacists are a key group that should be aware of the importance of nutrition. To highlight this opportunity/need, NNEdPro ran an essay competition entitled, “*The role of nutrition in pharmacy settings.”* The competition, the third in a series of annual NNEdPro essay competitions (12, 13), was open to those working in or studying pharmacy, and those attending the NNEdPro Summer School. The pharmacy winner was University of Herefordshire student, Eriata Abulu who presented ideas from her essay, as did Summer School student winner, Miranda Van Emmenis. Both competition winners and a panel of speakers highlighted that with additional training, pharmacists could fulfill a key role at the interface between the public and HCP to deliver evidence-based nutrition messages.

To demonstrate a specific example of how nutrition science integrates with pharmacists’ clinical role, Robert Winwood presented on drug nutrient interactions.

## Translating Novel Data on Polyphenols onto Practice

The emerging evidence regarding the benefits of polyphenols provided an interesting case study on how evidence-based nutrition that has proven clinical effectiveness can be translated into practice. To present this case, Daniele Del Rio (DDR) provided an introduction to polyphenols, while Christina Khoo set the scene by outlining Ocean Spray’s history and collaboration of research with universities, government agencies, and other companies to highlight evidence regarding the impact of cranberries on health.

A series of researchers outlined emerging evidence for polyphenols in health. Daniela Martini presented on requirements for scientific substantiation of health claims, indicating the benefits of using the three main pillars on which food claims are judged to provide guidance for the quality research required to have claims substantiated (14, 15). Donato Angelino presented evidence suggesting that polyphenols, in particular, proanthocyanidins, found in tea, cocoa, grapes, nuts, and berries, are beneficial for cognitive function. However, lack of robust biomarkers of dietary intake hampers progress in this field. Geoffrey Istas presented on the effects of polyphenols on vascular function in healthy men. Nathalie Tufenkji discussed cranberry as an alternative for prevention of urinary tract infections (UTIs), an infection that contributes to global spread of antibiotic resistance. Kalpana Gupta focused on the impact of cranberry on UTIs, the most common bacterial infection in women (16). KG’s research among women with recent history of UTI demonstrates a lowered number of symptomatic UTI episodes for those consuming cranberry juice (17), suggesting an alternative preventative measure.

## Implementation—Moving Research into Practice

### What Is Implementation Science and How Can It Improve Nutritional Care?

With the expanding evidence in nutrition research, knowing when and how to get that research into practice is key. Knowledge translation, implementation, behavior change, and communication of nutrition messages are important components when translating this research into practice. Celia Laur presented an example of an effective implementation project from the Canadian More-2-Eat project, which improved nutrition care in five Canadian hospitals. By working with hospital champions and support teams, and using a variety of implementation and behavior change strategies, all five hospitals successfully implemented nutrition screening, and a method of triaging at risk patients to receive appropriate care. A toolkit to support implementation is available online (18), and plans are underway to sustain change and spread nationally and internationally, including in the UK.

In the UK context, a barrier to addressing hospital malnutrition is the confusion and misunderstanding about who is responsible for malnutrition. Harrison Carter (HC) explained the need for HCP to understand how to get policy into practice, including how it may require a whole system/service change, such as staff contractual alterations and workforce agreements. He also mentioned the costs associated with implementation to improve hospital malnutrition as another barrier. Medical nutrition education at all levels was one practical recommendation to address policy implementation barriers.

Another UK example of an implementation project underway focused on nutritional management of cow’s milk allergy, currently, a neglected clinical area. The aim is to bring examples of best practice from hospital care into primary and community practice for this most common food allergy in infants and young children. Focus groups and interviews are being conducted to inform what needs to be done and how, with a theory of change model being created.

### Connecting Implementation, Education, and Behavior Change

Presenting accurate nutrition messages in a way that is easy to understand is another important aspect within public health. Anthony Warner, a well-known blogger with a “temperament,” presented on bad science and the truth about healthy eating. Faddish diets from insta-food stars thrive in a world that favors easy explanation. People are attracted to these easy explanations over real science, potentially making decisions harmful to health. In a world of “fake news” with many providing their opinion on nutrition, qualified nutrition professionals have an important role in providing information that is evidenced based and easily understood.

While messaging is important, we also need to change behavior. To effectively promote healthy eating behaviors, Suzanne Piscopo discussed how nutrition interventions should be based on behavior change theory, target specific audiences, and go beyond disseminating information to nurturing willingness to improve food intake and creating enabling environments (19). Laura Thomas provided an example of this, describing use of intuitive eating principles in practice to create changes in behavior (20). A broader perspective was taken by Victor Mogre who explained the evidence from across several countries regarding educational interventions to improve nutrition care competencies and delivery by doctors and other HCP (21). When it comes to medical nutrition education, there is far more in common across regions than there are differences. Needs assessments should inform the design of interventions in order to improve nutrition practice behavior.

### Bringing It All Together

Giles Yeo’s (GY) presentation, although focused on the genetics of body weight and several unique examples, provided a strong emphasis on nutrition communication, and transmission of that message from trusted sources. GY argued that academics have a duty to be passionate about the truth, but how the truth is told also matters.

Continuing with the translation of nutrition evidence into practice, another area with growing evidence is the use of probiotics in a care home environment, which was presented by Victoria Avery. This educational programme is used to increase care home staff knowledge of the potential benefits that probiotics can provide. Caryl Nowson provided another example of nutrition translation and the promotion of healthy aging through bringing together the training of medical, nursing, nutrition, and physical activity HCP. Many HCP include professional domains for lifestyle approaches to reducing disease, which enables nutrition in care of the older person to be made part of everyone’s business. To translate this approach into action, examples were provided including a project to increase muscle mass and strength in women living independently in retirement villages (22). The recommendation was that if all HCP curricula included nutrition competencies, skills learned would provide opportunities to develop real-world inter-disciplinary approaches for: disease prevention and management to identify nutritional risk; the importance of lifestyle to patients for health; and support nutritional self-management of patients.

## The Need for Open Data

There is a need for open nutrition data in all of its forms (i.e., source data, descriptive collated information, research intelligence, and evidence synthesis) to be available for relevant experts to access and analyze. A discussion, chaired by AL and led by Ruthie Musker, Nida Ziauddeen, and Glenys Jones, outlined the need for and use of open data. A significant challenge raised during the discussion was ethics; the need for consent from participants, through to challenges of ethical approval at organization level. For example, whether university ethics review boards would approve collection and future use as open data. Most people recognized the need for and benefits of open data and provided suggestions for how NNEdPro and GODAN can continue to advocate and develop systems for open data access, such as the use of trials registries and repositories, formats for standardizing the anonymization of subject data, and ways to link data sources.

## Conclusion

Nutrition education provides the means to connect the ever-expanding and changing body of research-based evidence to policy and practice. This year, the Summit focused more broadly on how to impact policy and the need to concentrate on the full food system from “pre-farm to post-fork.” Once it is clear that strong evidence exists and the timing is right for translation into policy and practice, implementation and behavior change techniques should be employed, targeting HCP and the public. In all areas within nutrition and health, clear and effective messages are needed along with strong measures of impact centered on an ultimate goal: to improve nutritional health and wellbeing for patients and the public with a longer term view to link nutritional interventions, both at population and individual levels, to measureable health outcomes.

## Ethics Statement

Ethical approval was not required for this article because the risk to summit participants was deemed minimal, and all speakers consented to the inclusion of their details and key messages.

## Author Contributions

JC and CL led on writing the manuscript, while JC, CL, HC, GJ, and SR were involved in editing and finalizing this article. SR provided senior oversight. All authors were involved in organizing the Summit. All speakers in Table 1 were provided the opportunity to review this article to ensure accurate reflection of their presentations, however, were not involved in writing.

## Conflict of Interest Statement

JC, CL, HC, GJ, and SR are core members of the NNEdPro group, which hosted the Summit.

## Footnotes

^1^Works with the science and technology of producing and using plants for food, fuel, fiber, and land reclamation.

^2^Agricultural extension is the application of scientific research and knowledge to agricultural practices through farmer education.
